# Race and Ethnicity and Comorbidities Among Medicare Beneficiaries With Young-Onset Dementia

**DOI:** 10.1001/jamanetworkopen.2025.28001

**Published:** 2025-08-20

**Authors:** Jiahui Dai, Toubby Chau, Maria M. Corrada, Spero M. Manson, Joan O’Connell, Luohua Jiang

**Affiliations:** 1 Department of Epidemiology & Biostatistics, Joe C. Wen School of Population & Public Health, Susan and Henry Samueli College of Health Sciences, University of California, Irvine, Irvine; 2 Department of Neurobiology, Weinberg College of Arts and Sciences, Northwestern University, Evanston, Illinois; 3 Department of Neurology, School of Medicine, University of California, Irvine, Irvine; 4 Centers for American Indian and Alaska Native Health, Colorado School of Public Health, University of Colorado Anschutz Medical Campus, Aurora

## Abstract

**Importance:**

Young-onset dementia (YOD), which develops before age 65 years, can bring additional challenges to patients and their caregivers. The prevalence of YOD and its associated comorbidities across US racial and ethnic populations remain unclear.

**Objective:**

To estimate the prevalence of YOD and examine associations between comorbidities and YOD among Medicare beneficiaries in various racial and ethnic groups.

**Design, Setting, and Participants:**

This cross-sectional study used data from January 2022 to December 2022. Data were extracted from the Centers for Medicare & Medicaid Services 2022 Medicare Beneficiary Summary File (MBSF) for Medicare beneficiaries aged 45 to 64 years with almost-continuous fee-for-service coverage in 2022. The MBSF Research Triangle Institute race code was used to identify Hispanic, non-Hispanic American Indian and Alaska Native, non-Hispanic Asian, non-Hispanic Black, and non-Hispanic White populations.

**Exposures:**

Comorbidities examined include diabetes, cardiovascular disease (CVD), hyperlipidemia, hypertension, depression, chronic kidney disease without end-stage kidney disease (ESKD), ESKD, liver disease, cancer, chronic obstructive pulmonary disease, traumatic brain injury (TBI), alcohol use disorder, drug use disorder, tobacco use disorder, and hearing loss.

**Main Outcomes and Measures:**

The outcome was YOD, defined as Alzheimer disease and related dementias (ADRD) occurring in individuals aged 45 to 64 years. YOD was identified using end-of-year indicators of ADRD from the MBSF 30 Chronic Conditions Data file. The age-standardized prevalence of YOD in 2022 was calculated using the direct standardization method, with White adults in our sample as the standard population across age groups.

**Results:**

In 2022, among a total of 2 189 231 Medicare beneficiaries aged 45 to 64 years, 71 092 (3.25%) received a diagnosis of YOD. Black adults had the highest age-standardized YOD prevalence (13 149 beneficiaries [3.76%]). Among beneficiaries aged 45 years and older with ADRD, the proportions of YOD were approximately 2 to 3 times higher among Black (13 149 beneficiaries [7.01%]), American Indian and Alaska Native (655 beneficiaries [6.49%]), and Hispanic (6090 beneficiaries [4.77%]) individuals compared with White (49 818 beneficiaries [2.89%]) and Asian (1380 beneficiaries [2.12%]) individuals. In addition, Black, Hispanic, and American Indian and Alaska Native adults had a higher prevalence of most examined comorbidities than White adults. Many comorbidities, such as TBI (odds ratio [OR] range, 8.80-14.77), depression (OR range, 4.19-5.12), and CVD (OR range, 3.54-5.49), were associated with YOD.

**Conclusions and Relevance:**

This cross-sectional study found that Medicare beneficiaries from minoritized racial and ethnic groups younger than 65 years experienced a higher burden of YOD. Improved early detection and enhanced care coordination may help address the needs of Medicare beneficiaries with YOD, especially those from minoritized racial and ethnic groups with multiple comorbidities.

## Key Points

**Question:**

What is the prevalence of young-onset dementia (YOD) and its associations with comorbidities among Medicare beneficiaries aged 45 to 64 years across racial and ethnic groups?

**Findings:**

In this cross-sectional study of 2 189 231 Medicare beneficiaries aged 45 to 64 years, 3.25% had YOD, with the highest prevalence among Black adults. YOD made up a significantly larger proportion of Alzheimer disease and related dementias among Black, Hispanic, and American Indian and Alaska Native adults aged 45 years and older compared with White and Asian populations and was associated with many comorbidities.

**Meaning:**

These findings suggest that improved early detection may help address the needs of Medicare beneficiaries with YOD, especially those from minoritized racial and ethnic groups with multiple comorbidities.

## Teaser

This cross-sectional study estimates the prevalence of young-onset dementia among Medicare beneficiaries aged 45 to 64 years from various racial and ethnic groups and its associations with comorbidities.

## Introduction

Alzheimer disease and related dementias (ADRDs) are most commonly observed in older adults, but they can also manifest in individuals younger than 65 years, which is referred to as young-onset dementia (YOD).^1^ Caregivers of patients with YOD face substantially more perceived difficulties and have access to fewer supportive resources compared with those caring for patients with late-life ADRD.^2^ Furthermore, proper YOD diagnosis is often delayed or misdiagnosed,^3,4^ potentially leading to more-aggressive disease progression.^5,6^ Thus, there is a growing recognition of the importance of the early identification of risk, timely diagnosis, and prevention of YOD.^7,8^

Numerous investigations have examined racial and ethnic disparities in ADRD among older adults in the US owing to their association with advanced age.^9^ Studies consistently report higher prevalence or incidence of ADRD among older non-Hispanic Black (hereafter, Black) and Hispanic adults compared with their non-Hispanic White (hereafter, White) counterparts.^10,11,12,13^ In contrast, reliable estimates of YOD prevalence and its associated comorbidities across US racial and ethnic populations are scarce. To our knowledge, only 1 study^14^ has estimated ADRD prevalence among Medicare beneficiaries of all ages, including those younger than 65 years. Although a few other studies have examined ADRD, cognitive impairment, or subjective cognitive decline among US adults younger than 65 years,^4,15,16,17,18,19^ findings from those studies are compromised by outdated data and reliance on unreliable methods such as self-reported outcomes.

The list of potentially modifiable risk factors for ADRD has recently been updated and includes high low-density lipoprotein cholesterol, hearing loss, depression, traumatic brain injury (TBI), physical inactivity, diabetes, smoking, hypertension, obesity, and excessive alcohol consumption during midlife.^20^ Previous studies^21,22,23,24,25,26^ have documented racial and ethnic disparities across most of these risk factors. For instance, in 2021, non-Hispanic American Indian and Alaska Native individuals (hereafter, American Indian and Alaska Native) were nearly twice as likely as White adults to have diabetes.^25^ Black, Hispanic, and American Indian and Alaska Native adults also experience higher rates of obesity than the White population.^21,22,23,24^ Black and American Indian and Alaska Native individuals have higher rates of hypertension.^21,23^ Given the elevated prevalence of risk factors for ADRD and their earlier occurrence among minoritized racial and ethnic populations, the anticipated increase in YOD burden among these populations poses a potentially considerable challenge for the US public health system in the next decades.

Medicare data include nearly all US adults aged 65 years and older, and a small proportion (7.7% in 2022) of US adults aged 45 to 64 years who qualify due to disabilities or serious health conditions.^27,28,29^ Consequently, younger Medicare beneficiaries likely have a substantially higher morbidity burden than the general US population of same ages. Using 2022 Medicare data, we estimated YOD prevalence and examined the associations between comorbidities and YOD among Medicare beneficiaries aged 45 to 64 years across racial and ethnic groups. We also estimated the proportion of YOD cases among all patients with ADRD.

## Methods

### Data Source and Study Population

In this cross-sectional study, we extracted data from the Centers for Medicare & Medicaid Services 2022 Medicare Beneficiary Summary File (MBSF), which includes information on age, sex, race and ethnicity, residence, health coverage, chronic conditions, and health care cost and utilization for Medicare beneficiaries enrolled in 2022.^30^ We classified beneficiaries as American Indian and Alaska Native, non-Hispanic Asian (hereafter, Asian), Black, Hispanic, and White using the MBSF Research Triangle Institute race code that is based primarily on self-reported race and ethnicity recorded in Social Security enrollment forms and is revised to better identify Hispanic, Asian American, or Native Hawaiian Pacific Islander individuals.^31,32^ Our study sample included adults aged 45 to 64 years who had Medicare Part A and B fee-for-service (FFS) coverage for 11 to 12 months or while alive in 2022 (eFigure in Supplement 1). The study was conducted through a Centers for Medicare & Medicaid Services Data Use Agreement (RSCH-2021-57502). The Colorado Multiple Institutional Review Board determined the study was exempt from review under category 4 of the Common Rule (45 CFR §46) and approved a full waiver of Health Insurance Portability and Accountability Act authorization. This study follows the Strengthening the Reporting of Observational Studies in Epidemiology (STROBE) reporting guidelines for cross-sectional studies.

### Covariates

We included demographic characteristics (age, sex, and race) and a comprehensive list of comorbidities. Guided by the 2024 Lancet Commission Report,^20^ we included diabetes, hyperlipidemia, hypertension, depression, TBI, alcohol use disorder, tobacco use disorder, and hearing loss. In addition, we incorporated other conditions shown to be associated with ADRD in prior research,^33,34,35,36,37,38,39,40^ including cardiovascular disease (CVD), chronic kidney disease without end-stage kidney disease (ESKD), ESKD, liver disease, cancer, chronic obstructive pulmonary disease, and drug use disorder. Most comorbidity measures were created on the basis of the end-of-year indicators from the MBSF 30 Chronic Conditions Data Warehouse (CCW) Chronic Conditions Segment,^30^ and Other Chronic or Potentially Disabling Conditions Segment. We defined any CVD as the presence of at least 1 of the following conditions: acute myocardial infarction, ischemic heart disease, heart failure, atrial fibrillation, peripheral vascular disease, or stroke (transient ischemic attack). ESKD was identified from Medicare enrollment records, as it is an eligibility criterion. For each condition, we created a binary indicator: 1 if the CCW end-of-year indicator equaled 3 (met claims criteria and had sufficient FFS coverage), and 0 otherwise. Most CCW end-of-year indicators were defined on the basis of *International Statistical Classification of Diseases and Related Health Problems, Tenth Revision,* codes from Medicare claims between January 2021 and December 2022, except stroke, which was defined using data from January to December 2022.

### Outcome Measurement

The outcome in this study was YOD, defined as ADRD occurring in individuals aged 45 to 64 years. YOD status was determined using the end-of-year indicator from the MBSF 30 CCW Chronic Conditions Segment, which identifies whether a beneficiary met ADRD claims criteria during January 2021 to December 2022. In CCW, the claims criteria were defined as the presence of at least 1 inpatient, skilled nursing facility, or home health agency claim, or 2 hospital outpatient or carrier claims with relevant diagnostic codes. The *International Statistical Classification of Diseases and Related Health Problems, Tenth Revision,* diagnostic codes used to identify ADRD, including codes for both Alzheimer dementia and non-Alzheimer dementia, are provided in eTable 1 in Supplement 1.^30^

### Statistical Analysis

The age-standardized prevalence of YOD in 2022 was calculated using the direct standardization method, with White adults in our sample as the standard population across age groups 45 to 49, 50 to 54, 55 to 59, and 60 to 64 years. The age-specific prevalence was determined by dividing the number of individuals in each age group with a diagnosis of ADRD (numerator) by the total number of beneficiaries in the corresponding age group (denominator). Prevalence was reported for the overall sample cohort as well as subpopulations stratified by race, ethnicity, age groups, and sex. We also calculated the proportion of adults with YOD among those aged 45 years and older with ADRD by dividing the number of YOD cases (numerator) by the total number of individuals aged 45 years and older with ADRD (denominator) for each racial and ethnic group. In addition, among Medicare beneficiaries aged 45 to 64 years, age, sex, comorbidities, and YOD status were presented as frequencies (percentages) and stratified by racial and ethnic groups. We used logistic regression models to determine age-adjusted bivariable and multivariable associations of demographic characteristics and comorbidities with YOD through unadjusted and adjusted odds ratios (aORs). Given the potential multicollinearity between several comorbidities in our study cohort (eg, >30% of individuals had both depression and tobacco use disorder) (eTables 2 to 5 in Supplement 1), we included only 1 condition from each set of collinear conditions in the multivariable models. Thus, our final multivariable models included age, sex, diabetes, any CVD, depression, TBI, alcohol use disorder, and hearing loss.

Sensitivity analyses were performed by incorporating CVD subtypes in the multivariable models. In addition, we used Poisson regression to estimate prevalence ratios for the associations of demographics and comorbidities with YOD. All statistical analyses were performed using SAS statistical software version 9.4 (SAS Institute), and 2-sided *P* < .001 was considered statistically significant.

## Results

A total of 2 189 231 FFS beneficiaries aged 45 to 64 years in 2022 (25 006 American Indian and Alaska Native, 46 901 Asian, 359 877 Black, 213 747 Hispanic, and 1 543 700 White) were included in this study, and 71 092 individuals (3.25%) had YOD (Table 1). The highest age-standardized YOD prevalence was observed among Black individuals (13 149 beneficiaries [3.76%]), followed by White individuals (49 818 beneficiaries [3.23%]), Asian individuals (1380 beneficiaries [3.02%]), Hispanic individuals (6090 beneficiaries [2.73%]), and American Indian and Alaska Native individuals (655 beneficiaries [2.69%]). Among all adults aged 45 years and older with ADRD, the proportions of those with YOD were 7.01% (13 149 beneficiaries) for Black individuals, followed by 6.49% (655 beneficiaries) for American Indian and Alaska Native individuals, 4.77% (6090 beneficiaries) for Hispanic individuals, 2.89% (49 818 beneficiaries) for White individuals, and 2.12% (1380 beneficiaries) for Asian individuals (eTable 6 in Supplement 1).

**Table 1.** Characteristics of Medicare Beneficiaries Aged 45 to 64 Years by Racial and Ethnic Group, 2022

Characteristic	Beneficiaries, No. (%)					
	American Indian or Alaska Native (n = 25 006)	Asian (n = 46 901)	Black (n = 359 877)	Hispanic (n = 213 747)	White (n = 1 543 700)	Full cohort (N = 2 189 231)
Beneficiaries with YOD, No.	655	1380	13 149	6090	49 818	71 092
Age-standardized YOD prevalence, %^a^	2.69	3.02	3.76	2.73	3.23	3.27
Age group, y						
45-49	3783 (15.13)	7175 (15.30)	53 499 (14.87)	31 851 (14.90)	204 322 (13.24)	300 630 (13.73)
50-54	5085 (20.34)	9582 (20.43)	70 666 (19.64)	41 479 (19.41)	286 795 (18.58)	413 607 (18.89)
55-59	6873 (27.49)	12 811 (27.31)	101 523 (28.21)	60 764 (28.43)	426 770 (27.65)	608 741 (27.81)
60-64	9265 (37.05)	17 333 (36.96)	134 189 (37.29)	79 653 (37.27)	625 813 (40.54)	866 253 (39.57)
Sex						
Male	12 705 (50.81)	23 693 (50.52)	181 723 (50.50)	113 313 (53.01)	807 079 (52.28)	1 138 513 (52.01)
Female	12 301 (49.19)	23 208 (49.48)	178 154 (49.50)	100 434 (46.99)	736 621 (47.72)	1 050 718 (47.99)
Comorbidities						
Hypertension	14 362 (57.43)	26 165 (55.79)	241 801 (67.19)	122 605 (57.36)	822 244 (53.26)	1 227 177 (56.06)
Hyperlipidemia	11 157 (44.62)	26 196 (55.85)	181 563 (50.45)	115 815 (54.18)	782 380 (50.68)	1 117 111 (51.03)
Depression	7917 (31.66)	11 904 (25.38)	98 573 (27.39)	66 857 (31.28)	596 378 (38.63)	781 629 (35.70)
Diabetes	10 272 (41.08)	18 029 (38.44)	133 385 (37.06)	85 959 (40.22)	417 325 (27.03)	664 970 (30.37)
Any cardiovascular disease	6899 (27.59)	12 743 (27.17)	114 934 (31.94)	59 990 (28.07)	420 865 (27.26)	615 431 (28.11)
Tobacco use disorder	6396 (25.58)	4841 (10.32)	66 241 (18.41)	28 187 (13.19)	368 386 (23.86)	474 051 (21.65)
Chronic obstructive pulmonary disease	3703 (14.81)	3387 (7.22)	43 840 (12.18)	19 747 (9.24)	273 469 (17.72)	344 146 (15.72)
Chronic kidney disease (no ESKD)	2764 (11.05)	5925 (12.63)	52 359 (14.55)	24 778 (11.59)	164 040 (10.63)	249 866 (11.41)
Drug use disorder	3621 (14.48)	2305 (4.91)	36 644 (10.18)	18 898 (8.84)	186 234 (12.06)	247 702 (11.31)
Liver disease	2648 (10.59)	4433 (9.45)	25 623 (7.12)	25 939 (12.14)	138 436 (8.97)	197 079 (9.00)
Any cancer	1147 (4.59)	2650 (5.65)	22 406 (6.23)	10 843 (5.07)	85 149 (5.52)	122 195 (5.58)
Alcohol use disorder	2228 (8.91)	1105 (2.36)	15 521 (4.31)	8772 (4.10)	78 228 (5.07)	105 854 (4.84)
ESKD	1860 (7.44)	4729 (10.08)	31 305 (8.70)	17 824 (8.34)	32 984 (2.14)	88 702 (4.05)
Hearing loss	1172 (4.69)	1747 (3.72)	9715 (2.70)	7759 (3.63)	61 520 (3.99)	81 913 (3.74)
Traumatic brain injury	314 (1.26)	387 (0.83)	2508 (0.70)	1614 (0.76)	18 851 (1.22)	23 674 (1.08)
≥2 Chronic conditions^b^	17 301 (69.19)	31 129 (66.37)	250 571 (69.63)	145 085 (67.88)	1 041 485 (67.47)	1 485 571 (67.86)

Abbreviations: ESKD, end-stage kidney disease; YOD, young-onset dementia.

^a^ The age-standardized prevalence of YOD in 2022 was calculated using White adults in our sample as the standard population based on the following age groups: 45 to 49, 50 to 54, 55 to 59, and 60 to 64 years.

^b^ Chronic conditions include hypertension, hyperlipidemia, depression, diabetes, any cardiovascular disease, tobacco use disorder, chronic obstructive pulmonary disease, chronic kidney disease without ESKD, drug use disorder, liver disease, any cancer, alcohol use disorder, ESKD, hearing loss, and traumatic brain injury.

Asian individuals were the youngest, with 15.30% (7175 beneficiaries) in the youngest (45-49 years) group, whereas White individuals were oldest. Approximately one-half of the individuals were male among American Indian and Alaska Native, Asian, and Black adults, whereas slightly more than half were male among Hispanic (113 313 beneficiaries [53.01%]) and White (807 079 beneficiaries [52.28%]) adults. Black adults had the highest prevalence of hypertension (241 801 individuals [67.19%]), followed by American Indian and Alaska Native, Hispanic, Asian, and White adults. Black adults also had the highest prevalence of any CVD (114 934 individuals [31.94%]). Asian (26 196 individuals [55.85%]) and Hispanic (115 815 individuals [54.18%]) adults had a relatively higher prevalence of hyperlipidemia than adults from other racial and ethnic groups. White adults (596 378 individuals [38.63%]) had the highest prevalence of depression, followed by American Indian and Alaska Native, Hispanic, Black, and Asian adults. American Indian and Alaska Native adults had a significantly higher prevalence of diabetes (10 272 individuals [41.08%]) and alcohol use disorder (2228 individuals [8.91%]), compared with other racial and ethnic groups (Table 1).

Table 2 presents the age-specific prevalence of YOD stratified by race and ethnicity, age, and sex. When stratified by age, we observed that YOD prevalence increased with age, with the highest prevalence in the oldest age group (60-64 years). Among those aged 60 to 64 years, the highest YOD prevalence was observed among Black (7066 of 134 189 individuals [5.27%), followed by White (26 970 of 625 813 individuals [4.31%]), Asian (690 of 17 333 individuals [3.98%]), Hispanic (3134 of 79 653 individuals [3.93%]), and American Indian and Alaska Native (328 of 9265 individuals [3.54%]) adults. Male individuals had higher prevalence of YOD than female individuals across all racial and ethnic groups. The largest sex disparities were observed among American Indian and Alaska Native adults with a difference of 0.5%, similar to the other minoritized racial and ethnic groups, which showed a sex difference around 0.4%.

**Table 2.** Prevalence of YOD Among Medicare Beneficiaries Aged 45 to 64 Years by Age Groups and Sex, 2022

Group	No. of beneficiaries with YOD/Total No. of beneficiaries (%)^a^					
	American Indian or Alaska Native	Asian	Black	Hispanic	White	Full cohort
Overall	655/25 006 (2.62)	1380/46 901 (2.94)	13 149/359 877 (3.65)	6090/213 747 (2.85)	49 818/1 543 700 (3.23)	71 092/2 189 231 (3.25)
Age group, y						
45-49	55/3783 (1.45)	109/7175 (1.52)	786/53 499 (1.47)	450/31 851 (1.41)	3028/204 322 (1.48)	4428/300 630 (1.47)
50-54	96/5085 (1.89)	222/9582 (2.32)	1681/70 666 (2.38)	828/41 479 (2.00)	6397/286 795 (2.23)	9224/413 607 (2.23)
55-59	176/6873 (2.56)	359/12 811 (2.80)	3616/101 523 (3.56)	1678/60 764 (2.76)	13 423/426 770 (3.15)	19 252/608 741 (3.16)
60-64	328/9265 (3.54)	690/17 333 (3.98)	7066/134 189 (5.27)	3134/79 653 (3.93)	26 970/625 813 (4.31)	38 188/866 253 (4.41)
Sex						
Male	364/12 705 (2.87)	746/23 693 (3.15)	6998/181 723 (3.85)	3453/113 313 (3.05)	26 771/807 079 (3.32)	38 332/1 138 513 (3.37)
Female	291/12 301 (2.37)	634/23 208 (2.73)	6151/178 154 (3.45)	2637/100 434 (2.63)	23 047/736 621 (3.13)	32 760/1 050 718 (3.12)
Male beneficiaries by age group, y						
45-49	33/2043 (1.62)	62/3817 (1.62)	405/27 554 (1.47)	276/17 110 (1.61)	1691/108 968 (1.55)	2467/159 492 (1.55)
50-54	50/2678 (1.87)	125/4994 (2.50)	887/36 345 (2.44)	474/21 999 (2.15)	3499/150 756 (2.32)	5035/216 772 (2.32)
55-59	98/3442 (2.85)	203/6557 (3.10)	1903/51 113 (3.72)	939/32 207 (2.92)	7217/221 724 (3.25)	10 360/315 043 (3.29)
60-64	183/4542 (4.03)	356/8325 (4.28)	3803/66 711 (5.70)	1764/41 997 (4.20)	14 364/325 631 (4.41)	20 470/447 206 (4.58)
Female beneficiaries by age group, y						
45-49	22/1740 (1.26)	47/3358 (1.40)	381/25 945 (1.47)	174/14 741 (1.18)	1337/95 354 (1.40)	1961/141 138 (1.39)
50-54	46/2407 (1.91)	97/4588 (2.11)	794/34 321 (2.31)	354/19 480 (1.82)	2898/136 039 (2.13)	4189/196 835 (2.13)
55-59	78/3431 (2.27)	156/6254 (2.49)	1713/50 410 (3.40)	739/28 557 (2.59)	6206/205 046 (3.03)	8892/293 698 (3.03)
60-64	145/4723 (3.07)	334/9008 (3.71)	3263/67 478 (4.84)	1370/37 656 (3.64)	12 606/300 182 (4.20)	17 718/419 047 (4.23)

Abbreviation: YOD, young-onset dementia.

^a^ The prevalence of YOD was calculated by dividing the number of individuals in each age group with Alzheimer disease and related dementias (numerator) by the total number of Medicare beneficiaries in the corresponding age group (denominator).

Table 3 presents the age-adjusted bivariable logistic regression analyses of sex and comorbidities on YOD, stratified by race and ethnicity. Male individuals had significantly higher odds of YOD across all racial and ethnic groups. All examined comorbidities, except cancer, were significantly associated with an increased odds of YOD among all racial and ethnic groups. TBI showed the greatest association with YOD (OR range, 8.80-14.77), followed by depression (OR range, 4.19-5.12) and any CVD (OR range, 3.54-5.49).

**Table 3.** Age-Adjusted Bivariable Logistic Regression of Comorbidities on Young-Onset Dementia by Racial and Ethnic Group, 2022^a^

Variable	OR (95% CI)						Interaction *P* value^b^
	American Indian or Alaska Native (n = 25 006)	Asian (n = 46 901)	Black (n = 359 877)	Hispanic (n = 213 747)	White (n = 1 543 700)	Full cohort (N = 2 189 231)	
Sex, male vs female	1.25 (1.07-1.46)	1.19 (1.07-1.32)	1.14 (1.10-1.18)^c^	1.17 (1.11-1.23)^c^	1.07 (1.05-1.09)^c^	1.09 (1.07-1.11)^c^	<.001
Comorbidities							
Diabetes	1.74 (1.49-2.03)^c^	1.83 (1.64-2.04)^c^	2.46 (2.38-2.55)^c^	1.95 (1.85-2.05)^c^	1.70 (1.67-1.74)^c^	1.84 (1.81-1.87)^c^	<.001
Any cardiovascular disease	3.93 (3.35-4.61)^c^	4.02 (3.60-4.50)^c^	5.49 (5.28-5.71)^c^	4.33 (4.10-4.56)^c^	3.54 (3.48-3.61)^c^	3.90 (3.84-3.96)^c^	<.001
Hypertension	2.31 (1.92-2.77)^c^	2.58 (2.27-2.94)^c^	4.01 (3.78-4.24)^c^	2.88 (2.70-3.07)^c^	2.04 (2.00-2.08)^c^	2.29 (2.25-2.33)^c^	<.001
Hyperlipidemia	1.94 (1.65-2.28)^c^	2.12 (1.88-2.40)^c^	2.52 (2.42-2.62)^c^	2.30 (2.17-2.43)^c^	1.81 (1.78-1.85)^c^	1.96 (1.93-1.99)^c^	<.001
Depression	4.28 (3.63-5.03)^c^	4.47 (4.01-4.99)^c^	5.12 (4.94-5.31)^c^	4.78 (4.53-5.05)^c^	4.19 (4.10-4.27)^c^	4.31 (4.24-4.38)^c^	<.001
Chronic kidney disease (no ESKD)	2.18 (1.80-2.64)^c^	2.10 (1.85-2.38)^c^	2.25 (2.16-2.34)^c^	2.25 (2.11-2.39)^c^	2.31 (2.26-2.36)^c^	2.30 (2.26-2.34)^c^	.48
ESKD	1.41 (1.08-1.84)	1.06 (.89-1.26)	1.78 (1.69-1.87)^c^	1.50 (1.39-1.63)^c^	1.60 (1.52-1.69)^c^	1.62 (1.57-1.67)^c^	<.001
Liver disease	2.16 (1.77-2.62)^c^	2.05 (1.78-2.36)^c^	2.29 (2.18-2.41)^c^	1.83 (1.72-1.95)^c^	1.87 (1.82-1.91)^c^	1.91 (1.87-1.95)^c^	<.001
Any cancer	.79 (.54-1.17)	.98 (.78-1.23)	.97 (.91-1.04)	1.04 (.93-1.16)	1.03 (1.00-1.07)	1.02 (.99-1.06)	.54
Chronic obstructive pulmonary disease	2.17 (1.82-2.58)^c^	2.39 (2.06-2.77)^c^	2.25 (2.16-2.35)^c^	2.78 (2.61-2.96)^c^	1.77 (1.74-1.81)^c^	1.90 (1.87-1.93)^c^	<.001
Traumatic brain injury	14.77 (11.24-19.41)^c^	10.27 (7.98-13.22)^c^	9.90 (9.01-1.87)^c^	10.88 (9.61-12.31)^c^	8.80 (8.48-9.13)^c^	9.05 (8.76-9.35)^c^	<.001
Alcohol use disorder	3.89 (3.25-4.67)^c^	3.51 (2.83-4.36)^c^	3.00 (2.83-3.17)^c^	3.14 (2.89-3.41)^c^	3.01 (2.93-3.09)^c^	3.02 (2.95-3.09)^c^	<.001
Tobacco use disorder	1.55 (1.32-1.83)^c^	1.78 (1.54-2.06)^c^	1.69 (1.63-1.76)^c^	1.76 (1.65-1.88)^c^	1.31 (1.28-1.33)^c^	1.40 (1.38-1.42)^c^	<.001
Drug use disorder	1.87 (1.55-2.25)^c^	1.81 (1.48-2.22)^c^	1.78 (1.70-1.87)^c^	1.98 (1.84-2.13)^c^	1.61 (1.57-1.65)^c^	1.67 (1.64-1.70)^c^	<.001
Hearing loss	1.93 (1.46-2.55)^c^	1.99 (1.61-2.46)^c^	2.08 (1.92-2.25)^c^	1.97 (1.78-2.18)^c^	2.31 (2.23-2.38)^c^	2.21 (2.15-2.28)^c^	<.001

Abbreviations: ESKD, end-stage kidney disease; OR, odds ratio.

^a^ The age-adjusted model adjusts for age only, which is considered a strong confounder.

^b^ *P* values for the interactions between demographics, comorbidities, and racial and ethnic groups.

^c^ *P* < .001 denotes statistical significance.

Table 4 presents ORs from the multivariable logistic regression analysis for age, sex, and 6 comorbidities across different racial and ethnic groups. The positive associations between comorbidities and YOD were attenuated compared with the bivariable analyses. The highest aOR observed across examined comorbidities was for TBI among American Indian and Alaska Native adults (aOR, 9.68; 95% CI, 7.20-13.01), indicating that American Indian and Alaska Native adults with TBI had 9.68 times higher odds of having YOD than those without TBI. Depression was associated with greater than 3-fold the odds for YOD across all racial and ethnic groups (aOR range, 3.21 in American Indian and Alaska Native adults to 3.95 in Hispanic adults). CVD was associated with nearly 3 times the odds of YOD in the full cohort (aOR range, 2.69 in White adults to 3.83 in Black adults). Black adults had the greatest association between diabetes and YOD (aOR, 1.36; 95% CI, 1.31-1.41). White adults had the greatest association of hearing loss with YOD (aOR, 1.75; 95% CI, 1.70-1.82), compared with aORs ranging from 1.42 to 1.49. Multivariable models including various subtypes of CVD and are presented in eTable 7 in Supplement 1. Stroke (transient ischemic attack) demonstrated the greatest association with YOD compared with other CVD subtypes, with the highest aOR observed among Black adults (aOR, 3.91; 95% CI, 3.75-4.08). The prevalence ratio estimates from the Poisson models (eTable 8 in Supplement 1) were similar to the ORs from logistic regressions.

**Table 4.** Multivariable Logistic Regression of Comorbidities on Young-Onset Dementia by Racial and Ethnic Groups, 2022

Variable	OR (95% CI)					
	American Indian or Alaska Native (n = 25 006)	Asian (n = 46 901)	Black (n = 359 877)	Hispanic (n = 213 747)	White (n = 1 543 700)	Full cohort (N = 2 189 231)
Demographics						
Age (per 5 y)	1.40 (1.29-1.51)^a^	1.33 (1.26-1.41)^a^	1.47 (1.45-1.50)^a^	1.39 (1.35-1.42)^a^	1.35 (1.34-1.37)^a^	1.38 (1.37-1.39)^a^
Male vs female sex	1.24 (1.05-1.46)^a^	1.12 (1.00-1.26)	1.34 (1.29-1.40)^a^	1.31 (1.24-1.38)^a^	1.17 (1.15-1.19)^a^	1.21 (1.19-1.23)^a^
Comorbidities						
Diabetes	1.16 (.98-1.37)	1.12 (1.00-1.26)	1.36 (1.31-1.41)^a^	1.15 (1.08-1.21)^a^	1.10 (1.08-1.12)^a^	1.15 (1.13-1.17)^a^
Any cardiovascular disease	2.84 (2.38-3.38)^a^	3.30 (2.92-3.71)^a^	3.83 (3.67-3.99)^a^	3.21 (3.03-3.40)^a^	2.69 (2.64-2.74)^a^	2.91 (2.86-2.95)^a^
Depression	3.21 (2.70-3.81)^a^	3.72 (3.32-4.17)^a^	3.87 (3.72-4.02)^a^	3.95 (3.74-4.18)^a^	3.35 (3.28-3.42)^a^	3.51 (3.45-3.57)^a^
Traumatic brain injury	9.68 (7.20-13.01)^a^	6.74 (5.13-8.86)^a^	6.21 (5.60-6.88)^a^	7.05 (6.17-8.05)^a^	6.24 (6.00-6.49)^a^	6.35 (6.13-6.57)^a^
Alcohol use disorder	2.46 (2.03-3.00)^a^	1.87 (1.49-2.36)^a^	1.73 (1.63-1.84)^a^	1.83 (1.68-2.00)^a^	1.91 (1.86-1.97)^a^	1.88 (1.83-1.93)^a^
Hearing loss	1.49 (1.12-2.00)	1.48 (1.19-1.85)^a^	1.42 (1.31-1.55)^a^	1.42 (1.27-1.58)^a^	1.75 (1.70-1.82)^a^	1.66 (1.62-1.71)^a^

Abbreviation: OR, odds ratio.

^a^ *P* < .001 indicates statistical significance.

## Discussion

Our cross-sectional study observed a relatively high age-standardized prevalence of YOD, at 3.27% (71 092 individuals), among US Medicare beneficiaries aged 45 to 64 years, highlighting the necessity for further research that focuses on YOD among US Medicare beneficiaries and the general population younger than 65 years. The age-standardized prevalence of YOD was highest among Black individuals (3.76%) aged 45 to 64 years and was above or close to 3% in the other racial and ethnic groups.

A recent study^14^ using 2017 to 2019 Medicare FFS and Advantage data examined the prevalence of ADRD on the basis of various ADRD identification algorithms applied in the literature and found a prevalence of highly likely to possible ADRD among beneficiaries aged 45 to 64 years ranging from 2.4% to 3.8%, aligning with our finding of 3.27%. Using self-reported data from surveys and diagnostic codes from health service records, a recent review and meta-analysis^41^ reported a global age-standardized YOD prevalence of 0.12% among adults aged 30 to 64 years. Another review,^42^ based on similar types of data, indicated that the global age-specific prevalence of YOD in individuals younger than 65 years increased from 0.34% to 0.36% from 1990 to 2021. As expected, these global estimates of YOD prevalence are much lower than our estimates using Medicare data. Compared with the US general population aged 45 to 64 years, Medicare enrollees had a higher proportion of Black adults and a lower proportion of Asian adults. In addition, Medicare enrollees were significantly older (eTable 9 in Supplement 1).^29^ Although only 7.7% of adults aged 45 to 64 years enrolled in Medicare coverage in 2022, most qualified due to disability or specific conditions.^27,28^ Therefore, our study sample likely has a markedly higher morbidity burden than the general population of the same age. Thus, it is not surprising that the YOD prevalence estimated using Medicare data is substantially higher than global estimates.

Notably, among beneficiaries aged 45 years and older with ADRD, the proportion of individuals with YOD among Black, American Indian and Alaska Native, and Hispanic adults was approximately 2 to 3 times higher than that observed in Asian and White adults. This finding indicates that beneficiaries younger than 65 years from minoritized racial and ethnic groups might experience a particularly high burden of YOD. Disparities in life expectancy might partially explain the higher YOD proportion in Black and American Indian and Alaska Native adults.^43^ Another possible reason for this observation is that individuals from minoritized racial and ethnic groups had higher risk for the medical conditions that qualify them for Medicare enrollment younger than 65 years and, thus, had higher proportions of Medicare enrollment in this age range. Because only 7.7% of all US adults aged 45 to 64 years were enrolled in Medicare, compared with 95.3% of adults aged 65 years and older, we do not have complete data for YOD among all US adults. Consequently, the actual proportions of YOD among all patients with ADRD should be even higher than what are reported here. Indeed, a previous study^44^ of ADRD among all American Indian and Alaska Native adults who used Indian Health Service and Tribal health services found that 14% of ADRD cases were YOD. Similarly, a recent systematic review^42^ estimated that the proportion of YOD was around 20% globally. All these studies strongly suggest more clinical and research resources should be allocated to YOD, a condition that was traditionally treated as a rare disease and received little attention from both clinical and scientific community.

With respect to comorbidities, we found that most of them were significantly associated with an elevated odds of having YOD across all racial and ethnic groups. These findings align with those of a prior study estimating YOD prevalence and its associated comorbidities in a French population, although that study did not analyze racial and ethnic variations.^45^ Our study found that the prevalence of comorbidities and their associations with YOD both varied by race and ethnicity. We found that the prevalence of most comorbidities for YOD was higher among American Indian and Alaska Native and Black adults. American Indian and Alaska Native adults exhibited the highest prevalence of diabetes, tobacco use disorder, alcohol use disorder, TBI, and hearing loss, whereas Black adults had the highest prevalence of hypertension and any CVD in age-adjusted bivariable models. In terms of multivariable associations, we identified TBI as the comorbidity most strongly associated with YOD across all racial and ethnic groups, with American Indian and Alaska Native adults demonstrating the largest association. Black adults had the greatest associations of diabetes and any CVD with YOD compared with other racial and ethnic groups.

To the best of our knowledge, this study is one of the first to estimate YOD prevalence and comprehensively examine the associations of comorbidities with YOD for various racial and ethnic groups of US Medicare beneficiaries aged 45 to 64 years. In addition, our study benefits from a large sample size, diverse racial and ethnic populations, and an analysis of many important comorbidities for YOD.

### Limitations

Several limitations need to be acknowledged. First, because of the cross-sectional design, it remains unclear whether the examined comorbidities preceded YOD or vice versa. However, our future work will examine YOD onset using longitudinal data. Second, although basic demographic characteristics and multiple comorbidities were evaluated, data on factors related to many social determinants of health, lifestyle, environment, and other health conditions, such as obesity, were either unavailable or had a lower-than-expected prevalence. Third, previous studies^46,47,48,49^ found that Black and Hispanic adults are more likely to experience missed or delayed ADRD diagnoses than White adults in primary care settings. This underrecording in claims or electronic health record data may underestimate YOD prevalence, particularly among minoritized racial and ethnic groups. Furthermore, ADRD identification using 2 outpatient diagnostic codes may also result in diagnostic variation across racial and ethnic groups. However, a prior Medicare study^50^ identified ADRD using a similar algorithm and reported the sensitivity and specificity for ADRD identification were 79% and 88%, respectively, suggesting the potentially high validity of this algorithm. Fourth, we analyzed data for adults with Medicare FFS Parts A and B coverage to reflect more-comprehensive health status data. Thus, the results may not be generalizable to adults with Medicare Advantage or who do not have Part A or B coverage for 11 to 12 months of the year. In addition, Medicare beneficiaries younger than 65 years are individuals with disabilities or severe health conditions (eg, ESKD),^27,28^ making them not representative of the general US population aged 45 to 64 years. Therefore, our YOD prevalence estimates likely overestimate the national rate. Conversely, most must first qualify for Social Security Disability Income and receive benefits for about 2 years, a process requiring substantial time and resources.^27,28^ Consequently, only 7.7% of US adults aged 45 to 64 years are enrolled in Medicare, and the total number of 71 092 YOD cases identified here likely underestimate the true national burden. Improving data collection, expanding surveillance beyond Medicare, and raising awareness are essential to better assess the national burden of YOD. Furthermore, Medicare includes only 1 race and ethnicity measure, which may lead to misclassification for some beneficiaries.

## Conclusions

In conclusion, our results highlight the importance of recognizing YOD as a serious but often overlooked public health issue and emphasize the importance of preventing comorbidities associated with YOD. Early screening and detection of YOD with improved health care coordination services^51,52^ might be critical, particularly for younger minoritized racial and ethnic populations. Prior research underscores the value of early interventions for TBI, CVD, and depression to mitigate ADRD risk,^53,54,55^ whereas our study emphasizes the necessity of these interventions for middle-aged adults. In addition, addressing disease stigma, cultural taboos, low community awareness, and limited access to effective postdiagnosis treatments may help improve the care of patients with YOD.

## Notes

Supplement 1. **eFigure.** Flowchart of the Study Cohort Selection **eTable 1.** Valid *ICD-10* Codes Based on 30 CCW Chronic Conditions Algorithms to Identify Young-Onset Dementia **eTable 2.** Cross-Tabulation of Depression, Alcohol Use Disorder, Drug Use Disorder, and Tobacco Use Disorder, 2022 **eTable 3.** Cross-Tabulation of Alcohol Use Disorder and Drug Use Disorder, 2022 **eTable 4.** Cross-Tabulation of Alcohol Use Disorder and Tobacco Use Disorder, 2022 **eTable 5.** Cross-Tabulation of Drug Use Disorder and Tobacco Use Disorder, 2022 **eTable 6.** Medicare Beneficiaries Aged 45 Years and Older With Young Onset Dementia (YOD) or Alzheimer’s Disease and Related Dementias (ADRD), 2022 **eTable 7.** Multivariate Logistic Regression of Comorbidities With Cardiovascular Disease (CVD) Subtypes on Young Onset Dementia (YOD) by Racial/Ethnic Groups, 2022 **eTable 8.** Age-Adjusted Bivariate Log-Poisson Model of Comorbidities and Young Onset Dementia (YOD) by Racial/Ethnic Groups, 2022 **eTable 9.** Age, Sex, Race Distribution of Medicare Enrollees Aged 45-64 years in Our Study Compared to the General U.S. Population Aged 45-64 Years Using American Community Survey (ACS) Data in the Year 2022

Supplement 2.
Data Sharing Statement
