# Supplementary material for: Race and Ethnicity and Comorbidities Among Medicare Beneficiaries With Young-Onset Dementia
Source: JAMA Netw Open. 2025 Aug 20;8(8):e2528001. doi: 10.1001/jamanetworkopen.2025.28001 (PMC12368701; doi:10.1001/jamanetworkopen.2025.28001)
Supplement: Supplement 1. — eFigure. Flowchart of the Study Cohort Selection eTable 1. Valid ICD-10 Codes Based on 30 CCW Chronic Conditions Algorithms to Identify Young-Onset Dementia eTable 2. Cross-Tabulation of Depression, Alcohol Use Disorder, Drug Use Disorder, and Tobacco Use Disorder, 2022 eTable 3. Cross-Tabulation of Alcohol Use Disorder and Drug Use Disorder, 2022 eTable 4. Cross-Tabulation of Alcohol Use Disorder and Tobacco Use Disorder, 2022 eTable 5. Cross-Tabulation of Drug Use Disorder and Tobacco Use Disorder, 2022 eTable 6. Medicare Beneficiaries Aged 45 Years and Older With Young Onset Dementia (YOD) or Alzheimer’s Disease and Related Dementias (ADRD), 2022 eTable 7. Multivariate Logistic Regression of Comorbidities With Cardiovascular Disease (CVD) Subtypes on Young Onset Dementia (YOD) by Racial/Ethnic Groups, 2022 eTable 8. Age-Adjusted Bivariate Log-Poisson Model of Comorbidities and Young Onset Dementia (YOD) by Racial/Ethnic Groups, 2022 eTable 9. Age, Sex, Race Distribution of Medicare Enrollees Aged 45-64 years in Our Study Compared to the General U.S. Population Aged 45-64 Years Using American Community Survey (ACS) Data in the Year 2022 [file jamanetwopen-e2528001-s001.pdf]

Supplemental Online Content

Dai J, Chau T, Corrada MM, Manson SM, O'Connell J, Jiang L. Race and ethnicity and comorbidities among Medicare beneficiaries with young-onset dementia. *JAMA Netw Open.* 2025;8(8):e2528001. doi:10.1001/jamanetworkopen.2025.28001

eFigure. Flowchart of the Study Cohort Selection

eTable 1. Valid *ICD-10* Codes Based on 30 CCW Chronic Conditions Algorithms to Identify Young-Onset Dementia

eTable 2. Cross-Tabulation of Depression, Alcohol Use Disorder, Drug Use Disorder, and Tobacco Use Disorder, 2022

eTable 3. Cross-Tabulation of Alcohol Use Disorder and Drug Use Disorder, 2022

eTable 4. Cross-Tabulation of Alcohol Use Disorder and Tobacco Use Disorder, 2022

eTable 5. Cross-Tabulation of Drug Use Disorder and Tobacco Use Disorder, 2022

eTable 6. Medicare Beneficiaries Aged 45 Years and Older With Young Onset Dementia (YOD) or Alzheimer's Disease and Related Dementias (ADRD), 2022

eTable 7. Multivariate Logistic Regression of Comorbidities With Cardiovascular Disease (CVD) Subtypes on Young Onset Dementia (YOD) by Racial/Ethnic Groups, 2022

eTable 8. Age-Adjusted Bivariate Log-Poisson Model of Comorbidities and Young Onset Dementia (YOD) by Racial/Ethnic Groups, 2022

eTable 9. Age, Sex, Race Distribution of Medicare Enrollees Aged 45-64 years in Our Study Compared to the General U.S. Population Aged 45-64 Years Using American Community Survey (ACS) Data in the Year 2022

This supplemental material has been provided by the authors to give readers additional information about their work.

© 2025 Dai J et al. *JAMA Network Open*.

eFigure. Flowchart of the Study Cohort Selection

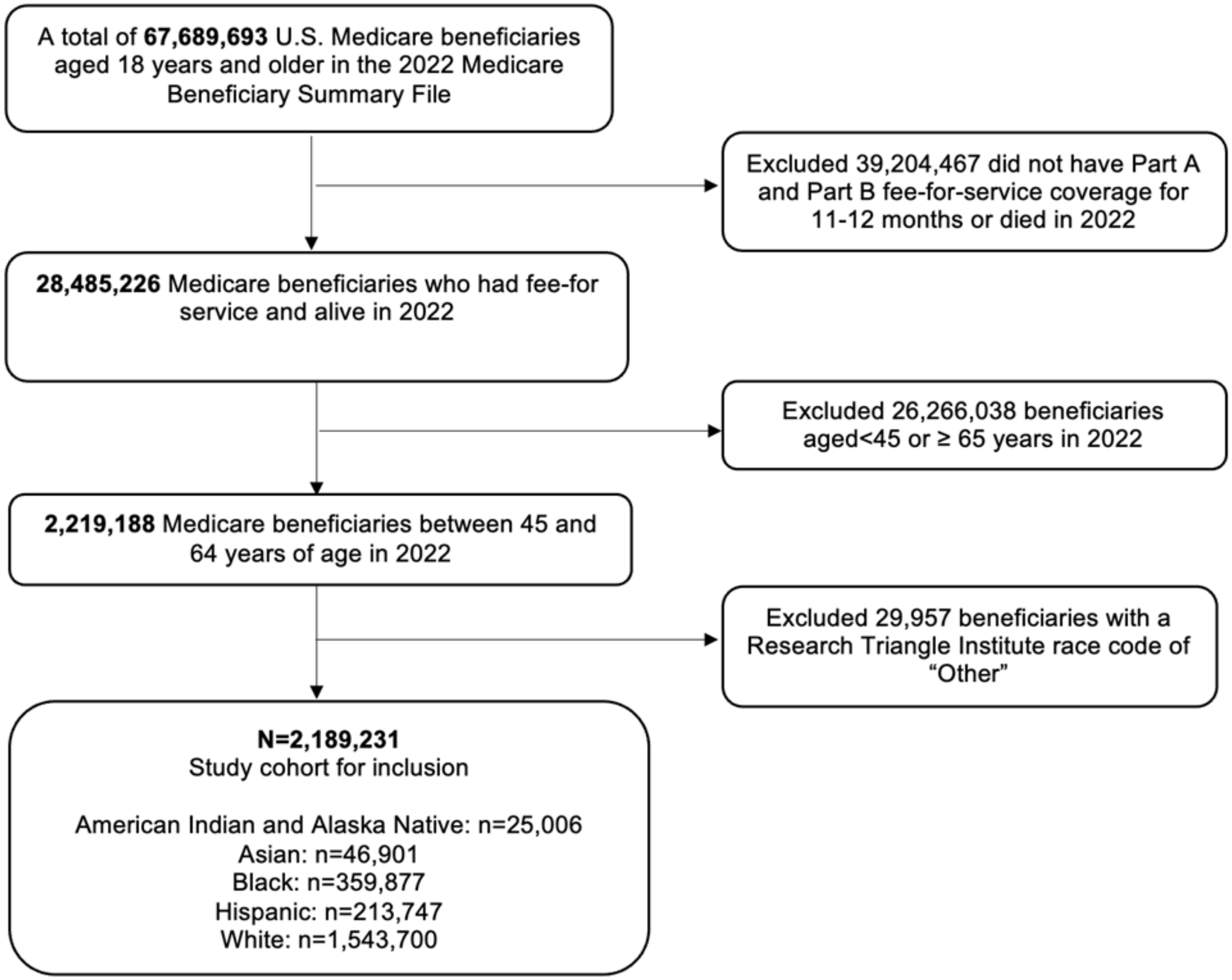

© 2025 Dai J et al. *JAMA Network Open*.

eTable 1. Valid *ICD-10* Codes Based on 30 CCW Chronic Conditions Algorithms to Identify Young-Onset Dementia

**Young-onset dementia**	**ICD-10 Diagnosis Codes**
Alzheimer's disease	G30.0, G30.1, G30.8, G30.9
Non-Alzheimer's Dementia	F01.50, F01.51, F01.511, F01.518, F01.52, F01.53, F01.54, F01.A0, F01.A11, F01.A18, F01.A2, F01.A3, F01.A4, F01.B0, F01.B11, F01.B18, F01.B2, F01.B3, F01.B4, F01.C0, F01.C11, F01.C18, F01.C2, F01.C3, F01.C4, F02.80, F02.81, F02.811, F02.818, F02.82, F02.83, F02.84, F02.A0, F02.A11, F02.A18, F02.A2, F02.A3, F02.A4, F02.B0, F02.B11, F02.B18, F02.B2, F02.B3, F02.B4, F02.C0, F02.C11, F02.C18, F02.C2, F02.C3, F02.C4, F03.90, F03.91, F03.911, F03.918, F03.92, F03.93, F03.94, F03.A0, F03.A11, F03.A18, F03.A2, F03.A3, F03.A4, F03.B0, F03.B11, F03.B18, F03.B2, F03.B3, F03.B4, F03.C0, F03.C11, F03.C18, F03.C2, F03.C3, F03.C4, F05, G13.8, G31.01, G31.09, G31.1, G31.2, G31.83, G94, R41.81

Abbreviations: ICD= International Classification of Diseases, Tenth Revision; CCW=Chronic Conditions Data Warehouse.

© 2025 Dai J et al. *JAMA Network Open*.

eTable 2. Cross-tabulation of Depression, Alcohol Use Disorder, Drug Use Disorder, and Tobacco Use Disorder, 2022

Depression	Alcohol use disorder	No.	Row %	Drug use disorder	No.	Row %	Tobacco use disorder	No.	Row %
No	Yes	40092	2.85	Yes	96402	6.85	Yes	228915	16.26
Yes	Yes	65762[a]	8.41[b]	Yes	151300[c]	19.36[d]	Yes	245136[e]	31.36[f]

[a]The number of individuals with depression who also had alcohol use disorder
[b]The proportion of individuals with alcohol use disorder among those with depression
[c]The number of individuals with depression who also had drug use disorder
[d]The proportion of individuals with drug use disorder among those with depression
[e]The number of individuals with depression who also had tobacco use disorder
[f]The proportion of individuals with tobacco use disorder among those with depression

eTable 3. Cross-tabulation of Alcohol Use Disorder and Drug Use Disorder, 2022

Alcohol use disorder	Drug use disorder	No.	Row %
No	Yes	200,887	9.64
Yes	Yes	46,815[a]	44.26[b]

[a]The number of individuals with alcohol use disorder who also had drug use disorder
[b]The proportion of individuals with drug use disorder among those with alcohol use disorder

eTable 4. Cross-Tabulation of Alcohol Use Disorder and Tobacco Use Disorder, 2022

Alcohol use disorder	Tobacco use disorder	No.	Row %
No	Yes	410,754	19.72
Yes	Yes	63,297[a]	59.80[b]

[a]The number of individuals with alcohol use disorder who also had tobacco use disorder
[b]The proportion of individuals with tobacco use disorder among those with alcohol use disorder

© 2025 Dai J et al. *JAMA Network Open*.

eTable 5. Cross-Tabulation of Drug Use Disorder and Tobacco Use Disorder, 2022

Drug use disorder	Tobacco use disorder	No.	Row %
No	Yes	333,237	17.16
Yes	Yes	140,814[a]	56.85[b]

[a]The number of individuals with drug use disorder who also had tobacco use disorder
[b]The proportion of individuals with tobacco use disorder among those with drug use disorder

eTable 6. Medicare Beneficiaries Aged 45 Years and Older with Young Onset Dementia (YOD) or Alzheimer's Disease and Related Dementias (ADRD), 2022

	AI/AN	Asian	Black	Hispanic	White	Full cohort
# of adult beneficiaries aged 45+	129,036	802,342	1,905,584	1,481,058	22,506,467	26,824,487
# of adult beneficiaries aged 45-64	25,006	46,901	359,877	213,747	1,543,700	2,189,231
% of 45-64 among adults aged 45+ [a]	19.38	5.85	18.89	14.43	6.86	8.16
# of those with ADRD aged 45+	10,093	65,237	187,517	127,755	1,726,586	2,117,188
# of YOD aged 45-64	655	1,380	13,149	6,090	49,818	71,092
% of YOD among adults with ADRD[‡] aged 45+ [b]	6.49	2.12	7.01	4.77	2.89	3.36

[a]The proportions of Medicare beneficiaries aged 45-64 years among those aged 45 and older was calculated by dividing the number of Medicare beneficiaries aged 45-64 (numerator) by the total number of Medicare beneficiaries aged 45 and older (denominator) for each racial/ethnic group
[b]The proportions of adults with YOD among those aged 45 and older with ADRD was calculated by dividing the number of YOD (numerator) by the total number of individuals aged 45 and older with ADRD (denominator) for each racial/ethnic group
Abbreviations: YOD; young-onset dementia; ADRD: Alzheimer's disease and related dementias

© 2025 Dai J et al. *JAMA Network Open*.

eTable 7. Multivariate Logistic Regression of Comorbidities With Cardiovascular Disease (CVD) Subtypes on Young Onset Dementia (YOD) by Racial/Ethnic Groups, 2022

	AI/AN (N=25,006)	Asian (N=46,901)	Black (N=359,877)	Hispanic (N=213,747)	White (N=1,543,700)	Full cohort (N=2,189,231)
	OR (95% CI)	OR (95% CI)	OR (95% CI)	OR (95% CI)	OR (95% CI)	OR (95% CI)
Demographics						
Age (Per 5 years)	1.41 (1.30, 1.53)*	1.35 (1.28, 1.43)*	1.47 (1.44, 1.50)*	1.40 (1.36, 1.43)*	1.37 (1.35, 1.38)*	1.38 (1.37, 1.40)*
Males vs. Females	1.23 (1.04, 1.45)	1.13 (1.01, 1.27)	1.32 (1.27, 1.37)*	1.28 (1.21, 1.35)*	1.18 (1.16, 1.21)*	1.21 (1.19, 1.23)*
Comorbidities						
Diabetes	1.12 (0.94, 1.34)	1.09 (0.96, 1.23)	1.30 (1.25, 1.36)*	1.06 (1.00, 1.13)	1.05 (1.03, 1.07)*	1.09 (1.08, 1.11)*
HD (AMI+IHD)	0.90 (0.72, 1.12)	1.06 (0.91, 1.23)	1.16 (1.11, 1.22)*	1.12 (1.05, 1.21)	0.95 (0.93, 0.97)*	1.00 (0.98, 1.02)
Other HD (HF+AF)	1.54 (1.25, 1.90)*	1.49 (1.28, 1.73)*	1.37 (1.31, 1.43)*	1.44 (1.34, 1.54)*	1.46 (1.42, 1.49)*	1.44 (1.41, 1.47)*
PVD	2.47 (2.02, 3.03)*	2.36 (2.05, 2.72)*	2.16 (2.07, 2.26)*	2.41 (2.26, 2.57)*	2.51 (2.46, 2.57)*	2.43 (2.39, 2.48)*
Stroke & TIA	2.65 (2.11, 3.33)*	3.25 (2.83, 3.73)*	3.91 (3.75, 4.08)*	2.99 (2.80, 3.20)*	2.70 (2.64, 2.78)*	3.00 (2.94, 3.06)*
Depression	3.14 (2.64, 3.74)*	3.58 (3.20, 4.02)*	3.58 (3.44, 3.72)*	3.79 (3.58, 4.01)*	3.25 (3.18, 3.32)*	3.37 (3.31, 3.42)*
TBI	9.48 (7.02, 12.79)*	6.38 (4.83, 8.43)*	5.58 (5.02, 6.21)*	6.86 (5.98, 7.86)*	5.92 (5.69, 6.16)*	5.98 (5.77, 6.20)*
Alcohol use disorder	2.54 (2.09, 3.10)*	1.89 (1.49, 2.39)*	1.82 (1.71, 1.94)*	1.87 (1.71, 2.05)*	1.92 (1.86, 1.98)*	1.90 (1.86, 1.95)*
Hearing loss	1.46 (1.09, 1.96)	1.39 (1.11, 1.74)	1.37 (1.26, 1.49)*	1.38 (1.24, 1.53)*	1.72 (1.66, 1.78)*	1.63 (1.58, 1.68)*

Note: *p<0.001 indicates statistical significance
Abbreviations: OR: odds ratio; CI: confidence interval; CVD: cardiovascular disease; YOD; young-onset dementia; HD: heart disease; AMI: acute myocardial infarction; IHD: ischemic heart disease; HF: Heart Failure; AF: Atrial Fibrillation; PVD: Peripheral vascular disease; TIA: Transient ischemic attack; TBI: Traumatic brain injury

© 2025 Dai J et al. *JAMA Network Open*.

eTable 8. Age-Adjusted Bivariate Log-Poisson Model of Comorbidities and Young Onset Dementia by Racial/Ethnic Groups, 2022

	AI/AN (N=25,006)	Asian (N=46,901)	Black (N=359,877)	Hispanic (N=213,747)	White (N=1,543,700)	Full cohort (N=2,189,231)
	RR (95% CI)	RR (95% CI)	RR (95% CI)	RR (95% CI)	RR (95% CI)	RR (95% CI)
**Demographics**						
Males vs. Females	1.24 (1.06, 1.45)	1.18 (1.06, 1.31)	1.13 (1.09, 1.17)*	1.16 (1.11, 1.23)*	1.07 (1.05, 1.08)*	1.09 (1.07, 1.10)*
**Comorbidities**						
Diabetes	1.71 (1.46, 2.00)*	1.79 (1.61, 1.99)*	2.37 (2.29, 2.45)*	1.91 (1.81, 2.01)*	1.67 (1.64, 1.70)*	1.79 (1.77, 1.82)*
Any CVD	3.76 (3.21, 4.40)*	3.82 (3.43, 4.26)*	5.10 (4.91, 5.31)*	4.11 (3.90, 4.33)*	3.36 (3.30, 3.42)*	3.69 (3.63, 3.75)*
Hypertension	2.26 (1.88, 2.71)*	2.52 (2.22, 2.86)*	3.85 (3.64, 4.07)*	2.80 (2.63, 2.99)*	1.99 (1.95, 2.03)*	2.23 (2.19, 2.27)*
Hyperlipidemia	1.90 (1.63, 2.23)*	2.08 (1.84, 2.34)*	2.43 (2.34, 2.53)*	2.24 (2.12, 2.38)*	1.78 (1.74, 1.81)*	1.92 (1.89, 1.95)*
Depression	4.07 (3.47, 4.78)*	4.20 (3.78, 4.68)*	4.70 (4.54, 4.87)*	4.51 (4.28, 4.76)*	3.97 (3.89, 4.05)*	4.07 (4.00, 4.13)*
CKD (no ESRD)	2.11 (1.76, 2.55)*	2.03 (1.80, 2.30)*	2.15 (2.07, 2.23)*	2.17 (2.04, 2.30)*	2.22 (2.17, 2.27)*	2.21 (2.17, 2.25)*
ESRD	1.40 (1.08, 1.81)	1.06 (0.89, 1.26)	1.72 (1.64, 1.81)*	1.48 (1.37, 1.60)*	1.57 (1.49, 1.65)*	1.59 (1.54, 1.64)*
Liver disease	2.09 (1.73, 2.53)*	1.99 (1.73, 2.28)*	2.18 (2.08, 2.29)*	1.79 (1.68, 1.90)*	1.81 (1.77, 1.86)*	1.85 (1.82, 1.89)*
Any Cancer	0.80 (0.54, 1.17)	0.98 (0.78, 1.22)	0.97 (0.91, 1.04)	1.04 (0.93, 1.15)	1.03 (1.00, 1.07)	1.02 (0.99, 1.05)
COPD	2.10 (1.77, 2.50)*	2.29 (1.99, 2.65)*	2.15 (2.07, 2.24)*	2.64 (2.49, 2.81)*	1.73 (1.70, 1.76)*	1.84 (1.81, 1.87)*
TBI	11.01 (8.69, 13.95)*	8.09 (6.48, 10.10)*	7.35 (6.78, 7.96)*	8.50 (7.63, 9.47)*	7.04 (6.81, 7.28)*	7.17 (6.96, 7.38)*
Alcohol use disorder	3.65 (3.06, 4.35)*	3.27 (2.66, 4.02)*	2.78 (2.63, 2.93)*	2.95 (2.72, 3.20)*	2.82 (2.75, 2.90)*	2.83 (2.77, 2.89)*
Tobacco use disorder	1.53 (1.31, 1.80)*	1.74 (1.51, 2.00)*	1.65 (1.58, 1.71)*	1.72 (1.62, 1.83)*	1.29 (1.27, 1.32)*	1.38 (1.36, 1.40)*
Drug use disorder	1.83 (1.52, 2.20)*	1.77 (1.45, 2.15)*	1.73 (1.65, 1.81)*	1.93 (1.80, 2.07)*	1.58 (1.54, 1.62)*	1.63 (1.60, 1.66)*
Hearing loss	1.88 (1.44, 2.46)*	1.93 (1.57, 2.36)*	1.99 (1.84, 2.14)*	1.91 (1.73, 2.11)*	2.21 (2.14, 2.28)*	2.12 (2.06, 2.18)*

Note: *p<0.001 indicates statistical significance

Abbreviations: RR: relative risk; CI: confidence interval; CVD: cardiovascular disease; CKD: chronic kidney disease; ESRD: end-stage renal disease; COPD: chronic obstructive pulmonary disease; TBI: Traumatic brain injury

© 2025 Dai J et al. *JAMA Network Open*.

eTable 9. Age, Sex, Race Distribution of Medicare Enrollees Aged 45-64 years in Our Study Compared to the General U.S. Population Aged 45-64 Years Using American Community Survey (ACS) Data in the Year 2022

	U.S. General Population[1] N (Col %)	All Medicare Enrollees N (Col %)	Medicare Fee-for-service (Our study) N (Col %)	% of Medicare enrollees	% of Medicare Fee-for-service Enrollees (Our study)
**Age**					
45–54	40,009,317 (48.9%)	1,842,073 (29.1%)	714,237 (32.6%)	4.6%	1.8%
55–64	41,736,980 (51.1%)	4,484,946 (70.9%)	1,474,997 (67.4%)	10.7%	3.5%
**Sex**					
Male	40,263,742 (49.3%)	3,116,589 (49.3%)	1,138,513 (52.0%)	7.7%	2.8%
Female	41,482,555 (50.7%)	3,210,428 (50.7%)	1,050,718 (48.0%)	7.7%	2.5%
**Race/Ethnicity**					
AI/AN	737,226 (0.9%)	48,259 (0.8%)	25,006 (1.1%)	6.6%	3.4%
Asian	5,022,729 (6.1%)	115,255 (1.8%)	46,901 (2.1%)	2.3%	0.9%
Black	9,744,877 (11.9%)	1,275,023 (20.2%)	359,877 (16.4%)	13.1%	3.7%
Hispanic	13,291,072 (16.3%)	798,720 (12.6%)	213,747 (9.8%)	6.0%	1.6%
White	52,954,033 (64.8%)	4,089,762 (64.6%)	1,543,700 (70.5%)	7.7%	2.9%
**Total**	81,746,297 (100.0%)	6,327,019 (100.0%)	2,189,231 (100.0%)	7.7%	2.7%

Abbreviation: AI/AN: American Indian and Alska Native.

[1]The statistics for the U.S. general population across racial/ethnic groups were from U.S. Census Bureau. 2022. 2022 American Community Survey data. Available from: https://data.census.gov/table/ACSDT1Y2022.B27006?q=Medicare;

© 2025 Dai J et al. *JAMA Network Open*.
