# Supplementary material for: Race and Ethnicity and Comorbidities Among Medicare Beneficiaries With Young-Onset Dementia
Source: JAMA Netw Open. 2025 Aug 20;8(8):e2528001. doi: 10.1001/jamanetworkopen.2025.28001 (PMC12368701; doi:10.1001/jamanetworkopen.2025.28001)
Supplement: Supplement 2. — Data Sharing Statement [file jamanetwopen-e2528001-s002.pdf]

Data Sharing Statement

Dai. Race and Ethnicity and Comorbidities Among Medicare Beneficiaries With Young-Onset Dementia. *JAMA Netw Open*. Published August 20, 2025.
doi:10.1001/jamanetworkopen.2025.28001

Data
Data available: No

Additional Information
Explanation for why data not available: The data came from the Centers for Medicare and Medicaid (CMS) and accessibility of data is regulated by the CMS.
